# A community-based feasibility randomized controlled study to test food-specific inhibitory control training in people with disinhibited eating during COVID-19 in Italy

**DOI:** 10.1007/s40519-022-01411-9

**Published:** 2022-06-06

**Authors:** Valentina Cardi, Valentina Meregalli, Elisa Di Rosa, Rossella Derrigo, Chiara Faustini, Johanna Louise Keeler, Angela Favaro, Janet Treasure, Natalia Lawrence

**Affiliations:** 1 Section of Eating Disorders, Department of Psychological Medicine, Institute of Psychiatry, Psychology & Neuroscience, King’s College London, London, UK; 2 Department of General Psychology, University of Padova, Via Venezia 8, 35131 Padua, Italy; 3 Department of Neurosciences, University of Padua, Padua, Italy; 4 Padua Neuroscience Center, University of Padua, Padua, Italy; 5 School of Psychology, University of Exeter, Exeter, Devon UK

**Keywords:** App, Binge eating, Bulimia nervosa, Depression, Food addiction

## Abstract

**Purpose:**

The aim of this study was to expand the evidence on the feasibility and impact of food-specific inhibitory control training in a community sample of people with disinhibited eating.

**Methods:**

Recruitment and data collection were conducted during the COVID-19 outbreak, in Italy. Ninety-four adult individuals with disinhibited eating were randomised to one of two conditions: App-based food-specific inhibitory control training or waiting list. Participants were assessed at baseline, end of intervention (2 weeks following baseline) and follow-up (one week later). The assessment measures included questionnaires about eating behaviour and mood.

**Results:**

Seventy-three percent of the sample reported a diagnosis of binge eating disorder, and 20.4% a diagnosis of bulimia nervosa. Retention rates were 77% and 86% for the food-specific inhibitory control training and the waiting list conditions, respectively. Almost half of the participants allocated to the training condition completed the “recommended” dose of training (i.e., 10 or more sessions). Those in the training condition reported lower levels of wanting for high-energy dense foods (*p* < 0.05), a trend for lower levels of perceived hunger (*p* = 0.07), and lower levels of depression (*p* < 0.05). Binge eating symptoms, disinhibition, wanting for high-energy dense foods, stress and anxiety were significantly lower at end of intervention, compared to baseline (*p* < .05).

**Conclusion:**

Findings corroborated the feasibility of food-specific inhibitory control training, and its impact on high-energy dense foods liking. The study expands the evidence base for food-specific inhibitory control training by highlighting its impact on perceived hunger and depression. The mechanisms underlying these effects remain to be clarified.

**Level of evidence:**

Level I, Evidence obtained from at least one properly designed randomized controlled trials; systematic reviews and meta-analyses; experimental studies.

## Introduction

Eating disorders are mental illnesses characterized by high mortality rates and disability [1]. These disorders are characterised by extreme eating behaviours, including undereating or overeating. Bulimia nervosa and binge eating disorder, in particular, are characterised by episodes of loss of control over eating, which might be followed by compensatory behaviours (e.g. dietary restraint, overexercise, purging behaviours; only in people with bulimia nervosa). Evidence-based, recommended psychological interventions, such as cognitive behavioural therapy or family-based therapy can be effective for some, although treatment follow-up rates remain disappointing (e.g. less than 50%) [2–4]. Furthermore, 50–70% of people with bulimia nervosa or binge eating disorder continue to experience binge eating or purging after receiving the most widely recommended evidence-based treatment for these conditions, enhanced cognitive behavioural therapy [5].

The COVID-19 pandemic has posed additional challenges to the treatment of eating disorders, due to a worldwide increase in the incidence of eating disorders behaviours in the community and a deterioration of symptoms in patients [6]. An area of particular relevance is the negative impact that the pandemic and social distancing have had on the use of unhealthy habits, such as the over overconsumption of unhealthy foods. Studies in the Italian population for example, have demonstrated a tendency to gain weight during lockdown [7] and also increased rates of emotional eating and binge eating [8, 9].

Emotional eating is defined as overeating in response to unpleasant emotional states (e.g. anger, sadness, guilt) [10], whereas binge eating is defined as the consumption of an objectively large amount of food in a discrete period of time, while perceiving loss of control and intense distress [11]. Both emotional eating and binge eating are characterised by disinhibited eating and often co-occur, with emotional eating precipitating binge eating episodes [12]. These behaviours are underlined by specific emotional and cognitive processes, including the over-evaluation of eating, weight and shape and their control, that have been described in the “transdiagnostic” model of eating disorders proposed by Fairburn and colleagues [13](Fairburn, Cooper and Shafran, 2002). These behaviours tend to be precipitated by negative affective states and might result from difficulties regulating emotions, including a tendency to react in an impulsive way to emotional states [14], as well impairments in inhibitory control (i.e., the ability to inhibit prepotent responses). There is initial evidence that deficits in emotion regulation and inhibitory control might be more accentuated in individuals with a greater proneness to show addictive-like responses to highly processed foods [15]. This behavioural phenotype has been described in the literature as “food addiction” [16], and is characterised by cravings for highly processed foods [17] and a greater tendency to use these foods to cope with negative emotions [18].

Increased negative affect and weakened inhibitory control have been documented in non-clinical populations as well, to explain disinhibited eating in adolescent [19, 20] and adult populations [21–23]. The findings in clinical, as well as non-clinical populations indicate that interventions aimed at improving inhibitory control and reducing negative affect might be helpful in targeting disinhibited eating, and that they might be particularly helpful among those with the food addiction phenotype.

Computerised and app-based trainings have been developed in recent years to improve general, and food-specific inhibitory control. These trainings adopt a modified version of the stop-signal task or of the go/no-go paradigms, in which high-calorie foods are repeatedly associated with response suppression and motor inhibition. In non-clinical populations, food-specific inhibitory control trainings have proven effective in reducing unhealthy eating, with interventions employing go/no-go paradigms producing the strongest effects [24, 25]. In a recent study, the use of an app-based inhibitory control training offered in addition to treatment as usual was associated with reductions in eating disorder psychopathology and high-energy dense food valuation in people with bulimia nervosa or binge eating disorder, compared to treatment as usual alone [26]. These findings mirror previous evidence demonstrating the association between the use of a computerised based food-specific inhibitory control training and moderate-to-large reductions in binge eating frequency, eating disorder psychopathology, and high-energy dense food valuation compared to general inhibitory control training in patients with binge eating symptoms [27].

The present study was aimed at expanding these findings at a time characterised by high perceived stress and disruptions of clinical services, the COVID-19 pandemic. The feasibility and efficacy of the app-based food-specific inhibitory training used by Keeler et al. [26] were tested during lockdown in Italy, in adults reporting overeating. Based on previous findings, the hypothesis was that food-specific inhibitory training would be associated with a greater reduction in binge eating episodes, compared to a waiting list. Between-group differences in changes in binge eating-related processes (i.e., high-energy dense food liking and wanting, cognitive restraint, disinhibition and hunger), and depression, anxiety and stress were also evaluated. Finally, the impact of food addiction on changes in binge eating frequency and eating-related processes was assessed.

## Methods

### Participants

Participants were recruited through flyers and informative materials published on social media, and through referrals from private nutritionists, non-profit organizations, and eating disorder clinical services. Inclusion criteria and exclusion criteria were assessed through a self-report screening form. Inclusion criteria included the following: 18 years old or older, fluent in Italian, owning a mobile device, and reporting episodes of binge-eating (defined as eating an objectively large amount of food in a discrete period of time, while experiencing a sense of lack of control and intense associated distress) or overeating (defined as episodes of eating more than is necessary to sustain oneself or that is physically comfortable [28]) over the previous three months. Exclusion criteria were a diagnosis of Anorexia Nervosa, a Body Mass Index (BMI) lower than 18.5, and the presence of a visual impairment that could not be corrected.

### Sample size estimation

Scholars recommend sample sizes between 24 and 50 participants for feasibility studies [29, 30]. Previous studies using the same application and version of the training found differences in food valuation of high-energy dense foods in a sample of 40 individuals. In this study, the goal was to recruit a minimum of 40 participants/group.

### Design and randomisation

**Fig. 1:**
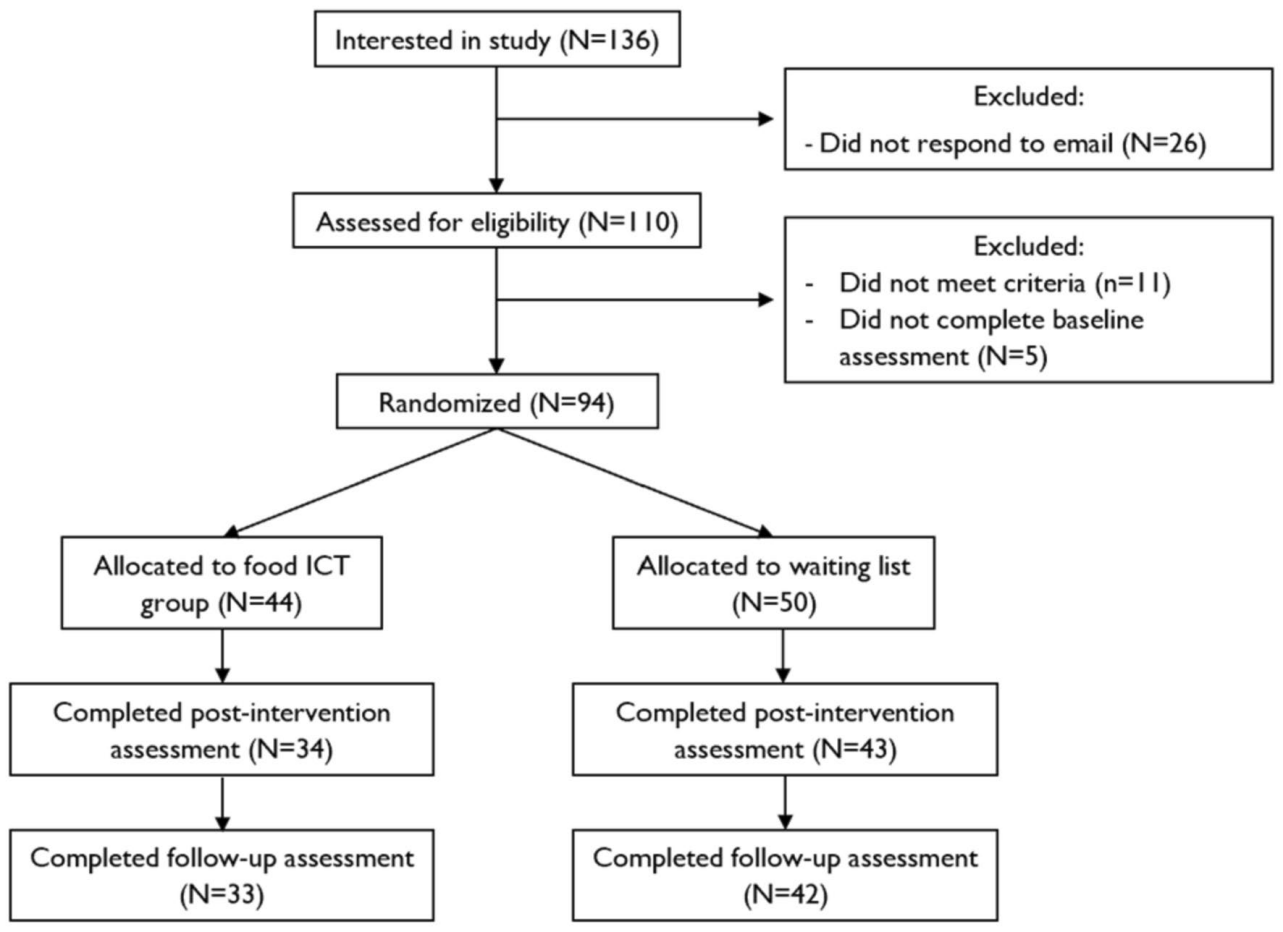
Consort diagram describing the flow of participation in the study

One-hundred and ten participants were screened for eligibility, and 94 individuals were included in the study. A random number generator (https://randomizer.org) was used to assign participants to the intervention (FoodT training; *N* = 44) or the control (Waiting list, *N* = 50) condition. The Consort Diagram (Fig. 1) describes the flow of participation in the study.

### Food-specific go/no-go training (FoodT)

**Fig. 2:**
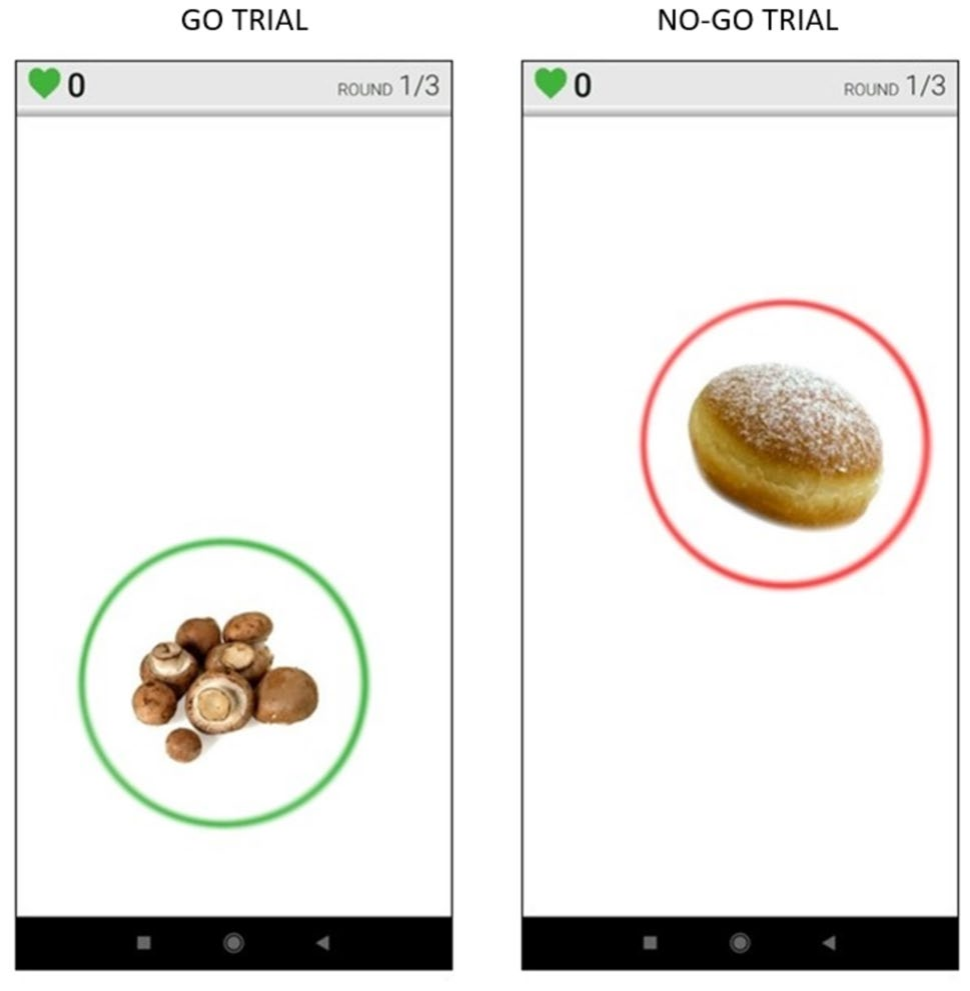
Examples of “go” (low-energy dense food, green circled) and “no go” stimuli (high-energy dense food, red circled) presented by the FoodT App, during the training

The food-specific go/no-go training was delivered through the FoodT App, a mobile application developed at the University of Exeter [31]. Participants were invited to complete at least 10 training sessions during a period of  two weeks. Each training session consisted of three blocks, and it lasted about 5 min. During each block, 32 images were individually presented on the screen for 1500 ms, with an interstimulus interval of 500 ms. Presented images included 8 low-energy dense foods (e.g. fruits, vegetables, and rice cakes), 8 high-energy dense foods (e.g. chocolate, cake, crisps), and 16 neutral objects (e.g. stationery, clothing). One hundred ms after picture presentation, a red or green circle appeared around the image. Participants were required to tap the image on the screen when the object was circled in green (“go” trials), and to inhibit the response when the object was circled in red (“no-go” trials). Low-energy and high-energy dense food pictures were always paired with “go” and “no-go” cues, respectively, while neutral objects were paired with either “go” or “no-go” cues (each 50% of times). Participants received feedback on their mean accuracy and reaction time at the end of each block. We analysed training task performance data to check that participants were accurately engaged in the training and showed evidence of learning the target stimulus–response associations (a ‘manipulation check’). Participants could choose up to three categories of high-energy dense foods to include in the training. Figure 2 provides examples of “go” and “no-go” stimuli.

### Assessment

#### Demographic variables

At baseline, participants completed a demographic questionnaire, which included questions on age, gender, weight, height, nationality, years of education, profession, current/previous mental health or medical problems and use of psychiatric medications.

#### Measures of eating behaviour and mood

At baseline and end of intervention participants completed the following scales: (1) the Binge Eating Scale [32], a 16-item scale for the assessment of binge eating behaviour (Cronbach’s alpha in this study = 0.88), (2) the Three Factors Eating Questionnaire (TFEQ) [33], a 51-item scale for the assessment of three dimensions of eating behaviour: Cognitive Restraint (Cronbach’s alpha in this study = 0.87), Disinhibition (Cronbach’s alpha in this study = 0.69), and Hunger (Cronbach’s alpha in this study = 0.75), (3) the Yale Food Addiction Scale (YFAS) [16], a 25-item scale for the assessment of food addiction (Cronbach’s alpha in this study = 0.97), and (4) the Depression Anxiety Stress Scales—Short Version (DASS-21) [34], a 21-item scale for the assessment of Depression (Cronbach’s alpha in this study = 0.84), Anxiety (Cronbach’s alpha in this study = 0.71), and Stress (Cronbach’s alpha in this study = 0.85). Moreover, participants’ levels of “liking” and “wanting” of 30 low- and high-energy dense foods were assessed using a visual analogue scale ranging from 0 to 10. The food items were different from those used in the training but belonged to the same categories (see https://osf.io/c8z6x/for the images, taken from [35]). All the measures, except for the TFEQ were collected also at follow-up.

### Procedure

After providing written consent, participants completed the baseline questionnaires via the online platform Qualtrics. Subsequently, they were randomly assigned to the food-specific training condition or to the waiting list condition. Participants assigned to the training condition received an email with instructions to download and use the FoodT application and were encouraged to complete at least ten training sessions in the following 2 weeks. These indications are based on findings and patient feedback from previous trials (i.e., eight sessions as minimum recommended dose of training, need for shorter time window to use the app compared to the  four weeks previously suggested [26, 27]. The contents of the App were in English, but participants were provided with an explanatory video to describe and demonstrate its usage. At the end of the two-week period (end of intervention), and  one week later (follow-up), they completed the same questionnaires completed at baseline, with the exception of the demographic questionnaire. Participants assigned to the waiting-list condition received instructions on how to use the FoodT application following completion of the follow-up assessment.

### Statistical analyses

Generalised linear mixed models were calculated using the lme4 package [36] in R 3.6.1. A model was calculated for each of the following dependent variables: total score of the BES; Cognitive Restraint subscale, Hunger subscale, and Disinhibition subscale of the TFEQ; Anxiety, Stress and Depression subscales of the DASS-21; and food liking and wanting for high-dense energy foods. The factors Group, Time (baseline, end of intervention, follow-up) and the interaction between Group and Time were included as fixed factors in the models. The categorial variable related to the possible presence of food addiction (Food Addiction) was included in the models calculated for the BES, TFEQ subscales, food liking and wanting. Participants’ identity was included as a random factor to control for repeated measurements of the same subject in all models performed.

The significance of the full model was established by comparing this model with the model that included only the random factor (null model) using the likelihood ratio test. The model fit and the over‐dispersion were checked using the DHARMa 0.3.3.0 package [37]. The significance value of each factor was derived using the “drop1” function [38]. The *multcomp* package [39] was used to derive significance values for Group by Time interactions (*lsmeans* function) and for the post hoc comparisons conducted on the Time variable (*glht* function). The Tukey post hoc correction was applied.

## Results

### Participants’ characteristics

Participants’ demographic and clinical characteristics are described in Table 1. There were no significant between-group differences in baseline characteristics (all *p* > 0.05). With the exception of four individuals, all participants identified themselves with the female gender (95.7%). Almost half (54.2%) were aged 35–54 years. Participants had completed on average 14 years of education. The mean body mass index (BMI) was 28.82 (SD = 7.67), at the upper end of the overweight category. Seventy-three percent of the sample reported a diagnosis of binge eating disorder and 20.4% a diagnosis of bulimia nervosa. The percentage of participants who were receiving psychological therapy at the time of participation was 38.4%; 29.1% were attending nutritional counselling and only a very small minority (5.8%) was taking psychiatric medication. The percentage of participants suffering from comorbid depressive symptoms or anxiety were 41.5%, and 45.7%, respectively.

**Table 1:** Participants’ demographic and clinical characteristics

Variable	Intervention condition	Control condition	All	Test statistic and *p* value
Age (*n*)				*X*^2^ (4) = 4.48 p = n.s
18–34	13	23	36	
35–54	27	24	51	
55–64	4	3	7	
Gender (*n*)				*X*^2^ (1) = 1.33 p = n.s
Male	3	1	4	
Female	41	49	90	
BMI (M, SD)	29.24 (7.63)	28.82 (7.67)	29.01 (7.61)	*t*(92) = − 0.26, p = n.s
Education (years M, SD)	14.27 (3.39)	14.96 (3.24)	14.63 (3.31)	*t*(92) = 1.0, p = n.s
Diagnosis of binge eating disorder (n)	33	35	68	*X*^2^ (1) = 0.46 p = n.s
Diagnosis of bulimia nervosa (n)	9	10	19	*X*^2^ (1) = 0.91 p = n.s
Eating disorder treatment in the past (n)	19	19	38	X^2^ (1) = 0.37 p = n.s
Current eating disorder treatment (n)				*X*^2^ (1) = 0.92 p = n.s
Psychological	17	16	33	
Nutritional	13	12	25	
Medication	3	2	5	
Comorbid depressive symptoms (n)	16	23	39	*X*^2^ (1) = 0.34 p = n.s
Comorbid anxiety (n)	22	21	43	*X*^2^ (1) = 0.43 p = n.s
Psychiatric medication (n)	6	4	10	*X*^2^ (1) = 0.37 p = n.s
Binge Eating Scale	20.70 (10.30)	22.80 (8.84)	21.81 (9.56)	*t*(92) = 1.06, p = n.s
TFEQ-Cognitive Restraint	12.36 (4.58)	11.92 (4.21)	12.12 (4.37)	*t*(92) = − 0.48, p = n.s
TFEQ-Disinhibition	11.70 (3.16)	12.08 (2.70)	11.90 (2.91)	*t*(92) = 0.62, p = n.s
TFEQ-Hunger	7.93 (3.30)	8.14 (3.31)	8.04 (3.29)	*t*(92) = 0.30, p = n.s
Liking for high-energy dense foods	5.44 (1.53)	5.29 (1.42)	5.36 (1.47)	*t*(92) = −0.49, p = n.s
Wanting for high-energy dense foods	4.37 (2.08)	4.51 (1.69)	4.45 (1.88)	*t*(92) = 0.35, p = n.s
Food addiction possible diagnosis (n)	23	25	48	*X*^2^ (1) = 0.82 p = n.s
DASS-Anxiety	5.54 (5.99)	6.96 (6.30)	6.29 (6.16)	t(92) = 1.11, p = n.s
DASS-Depression	12.77 (8.09)	13.68 (9.63)	13.25 (8.91)	t(92) = 0.65, p = n.s
DASS-Stress	17.50 (8.95)	18.68 (8.49)	18.12 (8.68)	t(92) = 0.49, p = n.s

Data described as frequencies (n) or mean (M) and standard deviation (SD)

*TFEQ* Three Factors Eating Questionnaire, *DASS* Depression Anxiety and Stress Scales. Test statistics and p values for the comparison between the Intervention and Control conditions

On average, participants reported moderate levels of binge eating symptoms [40], and high levels of attempts to restrain eating, disinhibition, and hunger on the TFEQ scale [41]. Just over half of the sample reported a possible diagnosis of food addiction based on the answers to the Yale Food Addiction Scale (i.e., three or more symptoms in addition to clinically significant impairment or distress) [16]. Participants reported moderate levels of anxiety, severe levels of depression and extremely severe levels of stress on the DASS-21 [42].

The group allocated to the FoodT condition completed on average 11 training sessions (min = 1, max = 30) over the two-week intervention period. Twenty-five participants completed ten or more sessions (56.8% of the sample). Forty participants completed a minimum of four sessions (two sessions/week, 90.9% of the sample). Training was completed to high levels of accuracy (Mean = 99.2%, SD = 0.66) and participants showed the expected learning of go/no-go contingencies during training. Paired-sample t tests indicated significantly faster reaction times for low-energy dense foods (Mean = 717.37, SD = 81.13) compared to filler items (Mean = 732.15, SD = 82.15; *t*(33) =  − 4.51, *p* < 0.0001), consistent with learning to “go” to low-energy dense foods. Participants also made fewer no-go errors to high-energy dense foods (Mean = 0.18%, SD = 0.4) compared to filler items (Mean = 0.5%, SD = 0.7; *t*(33) = 2.46, *p* = 0.02), suggesting they had learned to withdraw a motor response to unhealthy foods.

### Binge eating scale

The full model including all fixed factors was different from the null model (GLMM: *X*^2^ = 40.12, *df* = 6, *p* < 0.0001). The Group by Time interaction was not significant (*p* = 0.31), and therefore was removed from the model. The main effects of Group, Time, and Food Addiction were significant. Overall, those in the intervention condition reported lower scores (Mean = 18.24, SD = 10.08) compared to those in the control condition (Mean = 22.17, SD = 8.54). Those with a possible diagnosis of food addiction reported higher scores (Mean = 24.35, SD = 9.48) compared to those with no diagnosis (Mean = 16.39, SD = 7.59). Post-hoc tests indicated that participants reported significantly lower scores at end of intervention (Mean = 19.68, SD = 8.47) compared to baseline (Mean = 21.81, SD = 9.56, Estimate = − 2.04, SE = 0.71, z = -2.84; *p* = 0.012), and also at follow-up (Mean = 19.32, SD = 10.15) compared to baseline (Estimate =  −2.20, SE = 0.72, z = −3.06; *p* = 0.006). Scores at end of intervention and follow-up were not significantly different (Estimate = 0.16, SE = 0.74, z = 0.22; *p* = 0.97).

### Three factors eating questionnaire

For the Cognitive Restraint subscale, the full model including all fixed factors was not significantly different from the null model (GLMM: X^2^ = 1.24, *df* = 4, *p* = 0.87) and, therefore, no further analyses were conducted.

**Fig.3:**
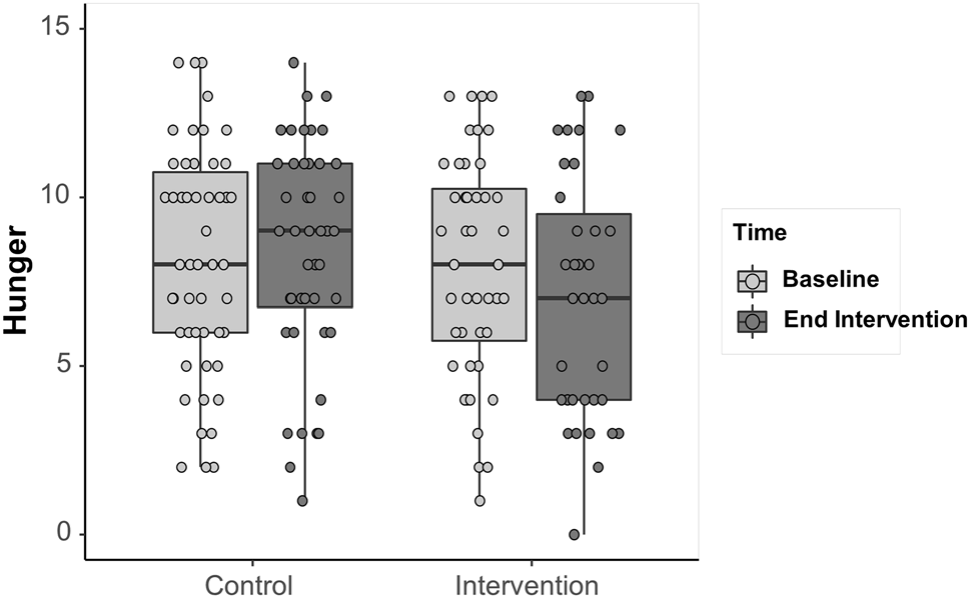
Differences between the intervention and control conditions, over time, in levels of hunger. The horizontal lines within the boxplot indicate the median. The boxes extend from the lower to the upper quartile and the whiskers indicate the interquartile range above the upper quartile (max) or below the lower quartile (min)

For the Hunger subscale, the full model including all fixed factors was significantly different from the null model (GLMM: X^2^ = 12.33, *df* = 4, *p* = 0.015). The interaction between Group and Time was significant; there was a trend for participants in the intervention condition to score lower at the end of the intervention (Mean = 6.97, SD = 3.61) compared to baseline (Mean = 7.93, SD = 3.30; Estimate = 0.92, SE = 0.37, *df* = 84.8, *t* ratio = 2.43, *p* = 0.07), whereas those in the control condition did not score significantly differently over time (Mean baseline = 8.14, SD = 31, Mean post = 8.39, Estimate = − 0.19, SE = 0.34, *df* = 83.3, *t* ratio = -0.55, *p* = 0.94; Fig. 3). Overall, those with a possible diagnosis of food addiction reported higher scores (Mean = 8.54, SD = 3.24) compared to those without a diagnosis (Mean = 7.27, SD = 3.40).

For the Disinhibition subscale, the full model including all fixed factors was significantly different from the null model (GLMM: *X*^2^ = 21.30, *df* = 4, *p* < 0.0001). The interaction between Group and Time was not significant (*p* = 0.12), and therefore, was removed from the model. The main effect of Group was not significant either. The main effect of Time was significant; participants reported significantly lower scores at end of intervention (Mean = 11.15, SD = 3.27) compared to baseline (Mean = 11.90, SD = 2.91). Those with a possible diagnosis of food addiction reported higher scores (Mean = 12.31, SD = 2.80) compared to those without a diagnosis (Mean = 10.80, SD = 3.20).

### High-energy dense food liking and wanting

**Fig. 4:**
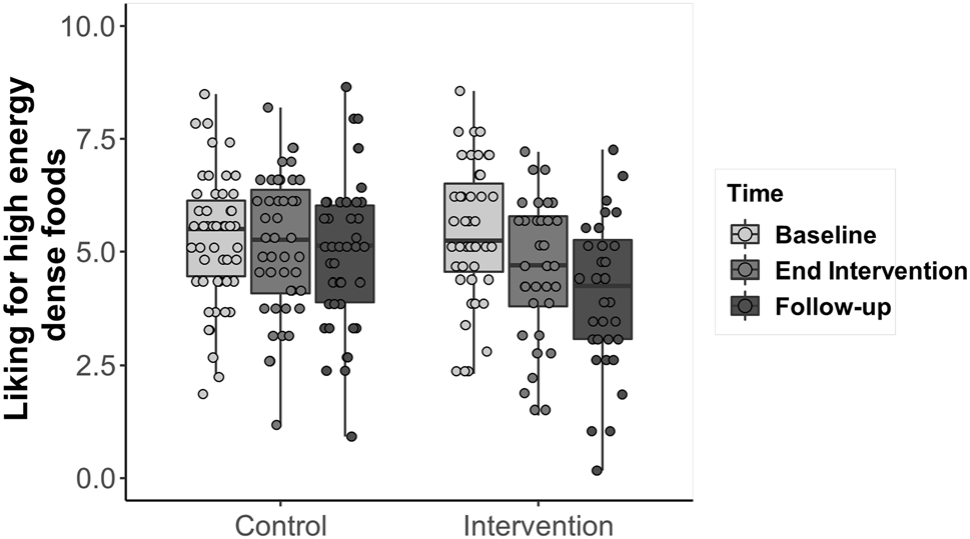
Differences between the intervention and control conditions, over time, in levels of liking for high-energy dense foods. The horizontal lines within the boxplot indicate the median. The boxes extend from the lower to the upper quartile and the whiskers indicate the interquartile range above the upper quartile (max) or below the lower quartile (min)

The full model for “food liking” was significantly different from the null model (GLMM: *X*^2^ = 45.17, *df* = 6, *p* < 0.0001). The interaction between Group and Time was significant. Participants in the intervention condition scored lower at the end of intervention (Mean = 4.58, SD = 1.54, Estimate = 0.70, SE = 0.18, *df* = 162, t ratio = 3.78, *p* = 0.002), and follow-up (Mean = 4.04, SD = 1.67; Estimate = 1.23, SE = 0.18, *df* = 162, t ratio = 6.51, *p* < 0.0001) compared to baseline (Mean = 5.44, SD = 1.53), whereas those in the control condition did not score significantly differently over time (Mean baseline = 5.29, SD = 1.42, Mean end of intervention = 5.15, SD = 1.52; Mean follow-up = 4.97, SD = 1.67; baseline vs. end of intervention: Estimate = 0.11, SE = 0.16, *df* = 160, *t* ratio = − 0.67, *p* = 0.98; end of intervention vs. follow-up: Estimate = 0.30, SE = 0.17, *df *= 161, t ratio = 1.77, *p* = 0.48; Fig. 4). The main effect of food addiction was not significant.

For the variable “food wanting”, the full model including all fixed factors was significantly different from the null model (GLMM: *X*^2^ = 21.25, *df* = 6, *p* < 0.0016). The interaction between Group and Time was not significant (*p* = 0.49), and therefore, was removed from the model. The main effect of Time was significant; participants reported significantly lower scores at end of intervention (Mean = 3.75, SD = 1.91; Estimate = 0.64, SE = 0.18, z =  −3.51 *p* = 0.0013) and follow-up (Mean = 3.71, SD = 1.95; Estimate =  −0.66, SE = 0.18, z = −3.57, *p* = 0.001), compared to baseline (Mean = 4.45, SD = 1.87). The difference between the end of intervention and follow-up scores was not significant (Estimate = 0.02, SE = 0.19, z = −0.11, *p* = 0.99). The main effects of Group and Food Addiction were not significant.

### Depression, anxiety and stress

**Fig. 5:**
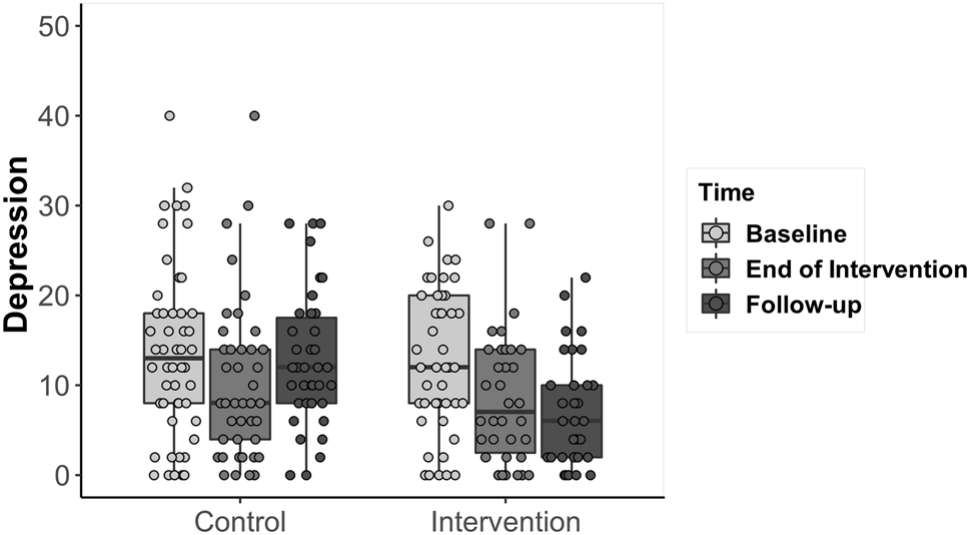
Differences between the intervention and control conditions, over time, in levels of Depression. The horizontal lines within the boxplot indicate the median. The boxes extend from the lower to the upper quartile and the whiskers indicate the interquartile range above the upper quartile (max) or below the lower quartile (min)

The full model for the variable Depression was significantly different from the null model (GLMM: *X*^2^ = 28.46, *df* = 5, *p* < 0.0001). The interaction between Group and Time was significant (*p* = 0.046). Participants in the intervention condition scored significantly lower at end of intervention compared to baseline (Mean baseline = 12.77, SD = 9.09, Mean end of intervention = 8.64, SD = 7.43; Estimate = 3.73, SE = 1.25, df = 163, t ratio = 2.49, *p* = 0.037) and lower at follow-up compared to baseline (Mean follow-up = 6.90, SD = 6.12, Estimate = 5.50, SE = 1.26, *df* = 163, *t* ratio = 4.36, *p* = 0.0003). Participants did not score significantly different at end of intervention compared to follow-up (estimate = − 1.76, SE = 1.30, df = 154, *t* ratio = − 1.35, *p* = 0.75). In the control condition, there were not significant differences between baseline (Mean = 13.68, SD = 9.63) and end of intervention scores (Mean = 10.23, SD = 8.82, Estimate = 3.08, SE = 1.13, *df* = 159, *t* ratio = 2.73, *p* = 0.07); between baseline and follow-up scores (Mean follow-up = 12.73, SD = 7.44, Estimate = 1.48, SE = 1.17, *df* = 160, *t* ratio = 1.26, *p* = 0.80); or between end of intervention and follow-up (Estimate = 1.60, SE = 1.20, df = 156, t ratio = 1.33, *p* = 0.76; Fig. 5).

For the Stress subscale, the full model including all fixed factors was significantly different from the null model (GLMM: *X*^2^ = 30.66, *df* = 5, *p* < 0.0001). The interaction between Group and Time was not significant (*p* = 0.16), and therefore was removed from the model. The main effect of Group was not significant either. The main effect of Time was significant. Participants reported overall higher scores at baseline (Mean = 18.12, SD = 8.68) compared to end of intervention (Mean = 14.23, SD = 8.32, Estimate = -3.70, SE = 0.84, z = − 4.39, *p* < 0.0001) and higher scores at baseline compared to follow-up (Mean follow-up = 14.27, SD = 8.61, Estimate = -3.72, SE = 0.85, z = − 4.35, *p* < 0.0001). There were not significant differences between scores at end of intervention and follow-up (Estimate = 0.02, SE = 0.88, z = 0.02, *p* = 1.0).

For the Anxiety subscale, the square root transformation was applied to match the normality assumptions. The full model including all fixed factors was significantly different from the null model (GLMM: X^2^ = 20.02, df = 5, *p* < 0.0012). The interaction between Group and Time was not significant (*p* = 0.34), and therefore, was removed from the model. The main effect of Group was not significant either. The main effect of Time was significant. Participants reported overall higher scores at baseline (Mean = 5.0, SD = 6.16) compared to end of intervention (Mean = 4.07, SD = 5.50, Estimate = − 0.37, SE = 0.09, z = − 3.76, *p* < 0.0001) and higher scores at baseline compared to follow-up (Mean follow-up = 4.37, SD = 5.64, Estimate = − 0.27, SE = 0.10, z = − 2.69, *p* < 0.019). No significant differences between end of intervention and follow-up scores were found (Estimate = − 0.009, SE = 0.10, z = − 0.91, *p* = 0.63).

Table 2 describes the parameters of the generalised linear mixed models calculated to assess between-group differences on eating behaviour, eating-related attitudes and depression, anxiety and stress over time.

**Table 2:** Parameters of the generalised liner mixed models calculated to assess between-group differences on eating behaviour, eating-related attitudes and depression, anxiety and stress over time

	Estimate	SE	t	*p*
TFEQ—Hunger				
Intercept	19.14	1.39	13.74	
Group	− 3.18	1.58	− 2.01	0.046
Time (baseline vs. end of intervention)	− 2.04	0.71	− 2.84	0.003
Time (baseline vs. follow-up)	− 2.20	0.72	− 3.06	
Food addiction	8.16	1.57	5.16	< 0.0001
TFEQ—Disinhibition				
Intercept	7.44	0.55	13.37	
Group	− 0.23	0.66	− 0.33	
Time	0.18	0.33	0.56	
Food addiction	1.39	0.63	2.20	0.028
Group x Time	− 1.11	0.50	− 2.21	0.028
Liking for high-energy dense foods				
Intercept	11.37	0.49	22.75	
Group	− 0.67	0.57	− 1.16	0.24
Time	− 0.66	0.20	− 3.23	0.0016
Food addiction	1.66	0.57	2.87	0.005
Wanting for high-energy dense foods				
Intercept	5.11	0.25	19.67	
Group	0.14	0.31	0.44	
Time (baseline vs. end of intervention)	− 0.11	0.16	− 0.68	
Time (baseline vs. follow-up)	− 0.30	0.16	− 1.80	
Food addiction	0.36	0.29	1.25	0.21
Group x Time (end of intervention)	− 0.59	0.24	− 2.40	0.001
Group x Time (follow-up)	− 0.93	0.25	− 3.70	
DASS-21 depression				
Intercept	4.35	0.30	14.32	
Group	− 0.37	0.34	− 1.09	0.27
Time (baseline vs. end of intervention)	− 0.64	0.18	− 3.51	0.0002
Time (baseline vs. follow-up)	− 0.66	0.18	− 3.57	
Food addiction	0.53	0.33	1.56	0.12
DASS-21 stress				
Intercept	13.68	1.14	11.92	
Group	− 0.90	1.67	− 0.54	
Time (baseline vs. end of intervention)	− 3.08	1.11	− 2.77	
Time (baseline vs. follow-up)	− 1.48	1.15	− 1.28	
Group X Time (baseline vs. end of int.)	− 0.65	1.66	− 0.39	0.046
Group x Time (baseline vs. follow-up	− 4.01	1.69	− 2.36	
DASS-21 anxiety				
Intercept	19.37	1.12	17.22	
Group	− 2.66	1.52	− 1.74	0.08
Time (baseline vs. end of intervention)	− 3.70	0.84	− 4.39	< 0.0001
Time (baseline vs. follow-up)	− 3.72	0.85	− 4.35	
Binge eating scale				
Intercept	2.64	0.14	18.19	
Group	− 0.37	0.20	− 1.85	0.07
Time (baseline vs. end of intervention)	− 0.37	0.09	− 3.76	0.0005
Time (baseline vs. follow-up)	− 0.27	0.10	− 2.69	

*TFEQ* Three Factors Eating Questionnaire, *DASS* Depression Anxiety and Stress Scales

## Discussion

The aim of this study was to expand findings on the impact of FoodT, a food-specific inhibitory training delivered through a mobile application, on eating behaviour, eating-related attitudes and psychological wellbeing in a community sample of people with disinhibited eating in Italy, during COVID-19. The use of FoodT was tested against a waiting list over 2 weeks, with an additional measurement taken 1 week later (follow-up). Comparing retention rates and number of sessions completed to those reported in a recent study testing the same form (App-based) and content of the training (food-specific) [26], retention rates at the end of the intervention were slightly lower (i.e., 77% vs 80%) in the intervention group, and overall lower than those in the control condition (86%). The average number of sessions completed was 11, and just over half of the participants completed at least 10 sessions over  two weeks, as had been recommended by the study team. When the number of sessions was lowered to eight in total (based on the feasibility threshold used in Keeler et al. [26]), then the number of participants meeting the criterion reached 68%. This is lower than the 80% found in Keeler et al. [26] Despite this, retention rates and number of sessions completed appear particularly remarkable considering that this study, unlike the previous ones, was conducted at a time characterised by high levels of uncertainty, distress, and disruptions to life routines. Also, although the sample was recruited from the community, over 70% self-reported a clinical diagnosis of binge eating disorder, and 20.4% self-reported a diagnosis of bulimia nervosa. Half of the sample had a possible diagnosis of food addiction and overall participants had moderate levels of binge eating. In terms of psychological wellbeing, at the time of testing participants were suffering from extremely high levels of stress, severe depressive symptoms, and moderate levels of anxiety. The characteristics of the sample mean that the conclusions which arise from this study might be relevant for clinical populations too (i.e., participants recruited from clinical services).

The intervention condition was associated with significantly lower levels of perceived hunger, high-energy dense food liking and depression symptoms, whereas it was not associated with significantly lower levels of binge eating symptoms. Some of these findings replicate previous ones i.e., reduction in high-dense energy foods valuation and no changes in binge eating, from a study conducted in patients receiving treatment for binge eating symptoms [26]. The finding related to a decrease in perceived hunger (a construct including both internally regulated and externally triggered hunger) is important, as previous experimental studies have proved the association between hunger, impaired response inhibition and an attentional bias towards foods [43]. Also, hunger has been associated with more intense cravings and a preference for food over non-food stimuli compared to satiation [44]. Thus, the reduction in perceived hunger is potentially important as it might have an effect on response inhibition, attentional bias to food and cravings, which are all associated to over consumption of highly palatable foods.

A similar argument could be made for the reduced liking of high-energy dense foods, as the preference for high-energy dense foods has been associated with a tendency to prefer these foods in a forced-choice task [45]. Interestingly though, this preferred choice did not translate into greater consumption of high palatable foods during a bogus taste test [45]. Consumption of food was not measured in this study, and therefore, it is not possible to conclude whether reduced hunger and/or liking of high-energy dense foods would translate to real-world eating behaviour. Whilst inhibitory control trainings have been associated with less consumption of food or alcohol compared to control conditions, in laboratory studies [46], real world effects are more mixed. For example, reduced food liking has been shown alongside weight loss or reduced intake in some studies [47, 48] but not others [49, 50].

Taken these findings together, one could suggest that food-specific inhibitory control trainings might impact on some key mechanisms of disinhibited hunger (e.g., perceived hunger and liking of high-energy dense foods) but that it may need to be combined with other forms of trainings (e.g., approach/avoidance trainings) in order to exert an impact on binge-eating. For example, a recent study comparing nicotine-avoidance training with nicotine-inhibition training in smokers found that avoidance training was more effective in reducing daily smoking in the short term [51].

In this study, those receiving inhibitory control training reported lower levels of depression symptoms over time. The assessment of mood is often neglected in the evaluation of motor response training procedures, and yet negative mood is strongly associated with unhealthy food consumption [27, 52, 53]. Future studies might seek to replicate this finding, and examine the mechanisms through which food-specific inhibitory control might exert a beneficial impact on mood. This study was conducted during COVID-19, and it is possible that engaging into a health-related activity might have given a sense of purpose to participants, boosting their mood. On the other hand, it is possible that there is something specific about inhibitory control training, which leads to improved mood, such as an increased sense of control or confidence over one’s behavioural choices.

### Clinical implications

This study adds to the literature discussing the potential of digital technology to enhance changes of unhelpful attitudes and behaviours in people with abnormal eating [54]. Findings should be considered preliminary and need further replication in larger randomised controlled clinical trials. Nevertheless, they seem to signal a positive impact of self-directed food-specific inhibitory control training on processes implicated in the maintenance of disinhibited eating, including perceived hunger, liking of high-energy dense foods and low mood. Future studies are warranted to establish whether adding guidance to the use of training might boost adherence and retention, as it has been previously suggested [55]. It is also warranted to investigate whether adding a food-specific inhibitory control training to standard treatment for eating disorders (binge eating disorder or bulimia nervosa) would boost the efficacy and effectiveness of standard treatment for these conditions. Furthermore, a large enough trial might provide indications as to how many sessions would constitute a “good enough” dose of training and collecting participants’ feedback would help improving the understanding of acceptability and mechanisms of the effect. Finally, it would be important to expand the investigation of app-based food-specific inhibitory control trainings to younger individuals, considering the increased frequency of admissions for intensive care in this age group [56].

### Strengths and limitations

The main strengths of this study are the use of an app-based training which was used before within the same patient group, thus enabling replication and broadening of findings, the use of a randomised controlled design, and the assessment of multiple outcomes, above and beyond binge eating symptoms. Limitations include the lack of a formal assessment of eating disorders and the lack of an objective measure of food consumption, higher than desirable attrition rates in the intervention condition, and the very low number of participants of non-female gender. Limitations also include the low power to establish whether participants in the intervention arm who were also receiving psychological, nutritional or pharmacological treatment, experienced greater benefit compared to those who were not receiving treatment. Finally, due to the limited resources available, longer follow-up times and structured interviews to gather participants’ feedback were not possible.

Despite these limitations, the study was conducted at a time of severe restrictions and many difficulties accessing clinical services in Italy. Findings appear encouraging and in line with previous evidence, which demonstrates the feasibility and benefits of using food-specific inhibitory control training.

### What is already known on this subject?

Food-specific inhibitory control training is associated with positive changes in health behaviour. Findings in people with disinhibited eating and a possible diagnosis of eating disorders are mixed.

### What this study adds?

This study tested the use of an app-based food-specific inhibitory control training in a community sample with disinhibited eating, during the COVID-19 Pandemic. Findings corroborated the feasibility of the training and expanded knowledge on its impact. In particular, this study highlighted the impact of training on perceived hunger and depression, opening interesting questions on the mechanisms which might explain these effects.

## Data Availability

Data are available on request to the corresponding author.
